# *Biomphalaria pfeifferi* (Gastropoda: Planorbidae) in Lake Malawi and Upper Shire River, Mangochi District, Malawi: Distribution, Genetic Diversity and Pre-Patent Schistosome Infections

**DOI:** 10.3390/tropicalmed8020126

**Published:** 2023-02-18

**Authors:** Mohammad H. Alharbi, Charlotte Condemine, Josie Hesketh, Sekeleghe A. Kayuni, Thomas M. Arme, John Archer, Sam Jones, E. James LaCourse, Peter Makaula, Janelisa Musaya, J. Russell Stothard

**Affiliations:** 1 Department of Tropical Disease Biology, Liverpool School of Tropical Medicine, Liverpool L3 5QA, UK; 2 Ministry of Health, Buraydah 52367, Saudi Arabia; 3 MASM Medi Clinics Limited, Medical Society of Malawi (MASM), Lilongwe P.O.Box 1254, Malawi; 4 School of Biodiversity, One Health & Veterinary Medicine, University of Glasgow, Glasgow G12 8QQ, UK; 5 Research for Health, Environment and Development (RHED), Mangochi P.O. Box 345, Malawi; 6 Malawi Liverpool Wellcome Trust Clinical Research Programme, Private Bag, Blantyre P.O. Box 30096, Malawi

**Keywords:** intermediate snail hosts, DNA barcoding, microsatellites, *Schistosoma mansoni*, intestinal schistosomiasis

## Abstract

In November 2017, *Biomphalaria pfeifferi*, the key intermediate host for *Schistosoma mansoni* in Africa, was first reported in Lake Malawi, Mangochi District. Two subsequent malacological surveys in 2018 and 2019 confirmed its lacustrine presence, as well as its presence along the Upper Shire River. These surveys provided sufficient specimens for analyses of the genetic structure and a transmission assessment for intestinal schistosomiasis. A total of 76 collected snails were characterized by a DNA sequence analysis of a 650 bp fragment of the mitochondrial cytochrome oxidase subunit 1 (*cox*1); by size fractionation of six fluorescently labelled microsatellite loci (Bgμl16, Bgμl, Bpf8, rg6, U-7, and rg9);by denaturing PAGE; and by detection of pre-patent *Schistosoma* infection by real-time PCR with a TaqMan^®^ probe. Five closely related *cox*1 haplotypes were identified, all present within a single location, with only one haplotype common across all the other locations sampled. No allelic size variation was detected with the microsatellites and all loci were monomorphic. Overall, the pre-patent prevalence of *Schistosoma* spp. was 31%, with infected snails found at several sampling locations. In this part of Lake Malawi, *Bi. pfeifferi* exhibits low genetic diversity and is clearly being exposed to the miracidia of *S. mansoni*, which is likely facilitating the autochthonous transmission of this parasite.

## 1. Introduction

The freshwater pulmonate snail *Biomphalaria pfeifferi* (Gastropoda: Planorbidae) is a key intermediate host for *Schistosoma mansoni* (Trematoda: Schistosomatidae) [1]. The latter helminth species is a parasitic blood fluke responsible for intestinal schistosomiasis. This fluke can be found across much of Africa and South America [1], and broadly speaking, the endemic zone of intestinal schistosomiasis is delineated by the underlying distribution of permissive snail populations. As the intermediate snail host populations change or vary, the actual or at-risk areas for disease transmission may also change; these dynamics, or fluctuations, are driven in part by ongoing and/or future climate change [2]. Whilst schistosomiasis transmission typically takes place within small water contact foci, even very large water bodies, such as Lake Malawi, are not invulnerable to environmental or biological transformations. For example, within this lake, there have been several significant ecological changes during the past four decades, most probably arising from environmental determinants, actions of humans, and the host–parasite evolution of snails and schistosomes [3].

Lake Malawi occupies about one-fifth of Malawi, representing a unique natural resource for water provision, fishing, agriculture, and international tourism [1]. In the 1980s, for the first-time, urogenital schistosomiasis started to be demonstrated among international tourists, with the later incrimination of sandy beaches with open waters as transmission foci, where deep water *Bulinus nyssanus* could be found [4]. Contrary to commonly held assumptions that *Bulinus globosus* was the only local host for *Schistosoma haematobium*, it was demonstrated experimentally and observed from natural infections that *Bu. nyassanus* was a permissive host. Indeed, several lacustrine factors influence the transmission of urogenital and, latterly, intestinal schistosomiasis within the lake. About 1000 fish species are estimated to be in the lake with many being molluscivorous [5]; however, with continuous over-fishing, fish predation was relaxed and thought to allow the expansion of snail populations, particularly the softer-shelled intermediate snail hosts of schistosomes [6]. At the same time, several environmental factors, either individually or in combination, are changing the lacustrine environment. There is increasing local pollution from an expanding local human population, reducing water quality, alongside the extraction of substantial amounts of lake water for irrigation purposes which run back into the lake creating more permanent water bodies around its periphery. These wet points would have otherwise dried out during the year and now, if colonized by snails, provide local refugia [5,6,7]. 

While urogenital schistosomiasis is considered the most common form of schistosomiasis along the Lake Malawi shoreline, there have been dramatic changes in the epidemiology of both forms of schistosomiasis locally [8]. This includes the detection of hybrid schistosomes within the *S. haematobium* group and the first report of *Bu. africanus* and *Bu. angolensis* within the lake, with currently unknown transmission potentials with *S. haematobium* [8,9]. More importantly, there was the first detection of *Bi. pfeifferi* within the lake in November 2017 [9,10]. Historically, *Bi. pfeifferi* was not considered endemic within the lake and is acting as a biological facilitator of a fulminating outbreak of intestinal schistosomiasis within Mangochi District [11]. Two later malacological surveys undertaken in 2018 and 2019 collected additional snail material sufficient for population genetic analyses. In this paper, we seek to determine the genetic structure of *Bi. pfeifferi*, alongside an assessment of pre-patent infection levels with *Schistosoma* spp. by molecular xenomonitoring.

## 2. Materials and Methods

### 2.1. Malacological Surveillance

**Figure 1:**
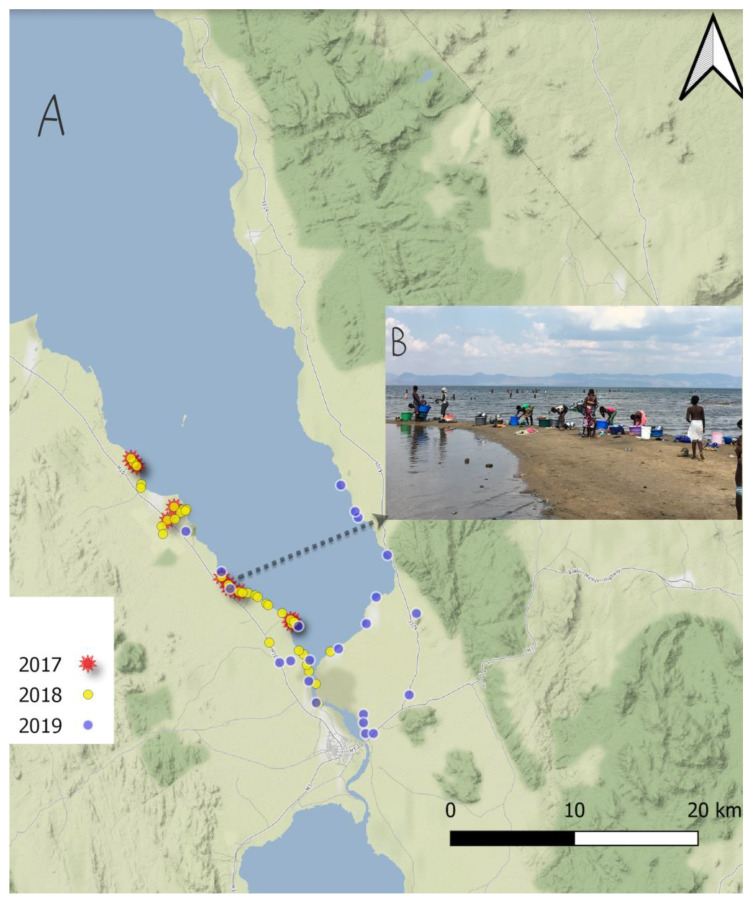
(**A**) The sample sites of visited locations in Mangochi District during each of the November 2017, May 2018, and May 2019 surveys. (**B**) Picture from a typical lakeshore site, displaying the extensive water contact by local people.

The malacological surveys comprised three directed surveys, pilot, exhaustive, and confirmation studies, carried out in 2017, 2018, and 2019, respectively, along the southern shoreline of Lake Malawi, and nearby water bodies, in Mangochi District (Figure 1). Based on the unexpected finding of *Biomphalaria* in the pilot study in 2017, we conducted a more exhaustive sampling study in May 2018 (after the rainy season) and visited 43 habitats in the southern region of the lake. One year after, in May 2019, we conducted a confirmation study, including 22 sites distributed in the southern and the eastern region of Lake Malawi. In all the surveys, we considered the collection of snails at known water contact sites, where human activities such as swimming, fishing and, washing take place (Figure 1B).

The average time for visiting each snail sampling site was 20 min, during which active searching using slotted scoops took place. According to the habitat type, the scoops were either long- or short-handled, depending on the water depth of the collection site. Then, all the collected snails were sorted by site and transported to the field laboratory. All the snails were kept in glass or plastic bottles in the dark for several hours before being exposed to light to stimulate cercarial shedding. Two days after the collection, this inspection was performed by visually checking the cercariae under the dissecting microscope. Finally, all the snails were preserved in 70% ethanol in universal glass bottles with aluminum caps and labelled with location code and date for transportation back to the Liverpool School of Tropical Medicine (LSTM), UK. Research authorizations were approved in the UK by the LSTM Research Ethics Committee (application 17-018) and in Malawi by the National Health Sciences Research Committee (1805).

### 2.2. Morphological Analyses, Sample Preparation and DNA Extraction

On arrival at the LSTM, all the samples collected in 2017, 2018, and 2019 were identified to genus level by morphological characteristics [1] and recounted per location prior to DNA extraction. Total genomic DNA was extracted from head–foot snail tissue, according to the methodology of Al-Harbi et al. (2022), utilizing the DNeasy Blood and Tissue Kit (Qiagen™, Hilden, Germany).

### 2.3. PCR and DNA Sequencing Analysis

A partial region of the mitochondrial cytochrome oxidase subunit 1 (*cox*1) was amplified using LC1490 and HCO2198 [12] forward and reverse primers, respectively, in a 25 μL PCR reaction for later sequence analysis. PCR was performed using Illustra puReTaq Ready-To-Go PCR beads (GE Healthcare), containing of 0.2 μM of forward and reverse primers, 2 μL of genomic DNA, and 21 μL of nuclease-free water. Successful amplification was verified by running 5 μL of PCR products across a 1% agarose gel for 40 min and visualizing a 650 bp length fragment. The PCR products that had successfully amplified the target region were then purified utilizing the MinElute PCR Purification Kit (Qiagen™, Hilden, Germany) according to the manufacturer’s instructions. The purified PCR products were then shipped to Source Bioscience (www.sourcebioscience.com, accessed on 1 October 2022), Cambridge, UK, for Sanger sequencing. All the sequence and chromatogram data retrieved were then analyzed and trimmed using Geneious 11.1 (Geneious). Using the NCBI database (https://blast.ncbi.nlm.nih.gov/Blast.cgi, accessed on 12 February 2022), all the trimmed sequences were individually subjected to a nucleotide BLAST search to speciate each isolate and investigate any genetic variation from previously deposited sequences. 

### 2.4. Microsatellite Markers Used to Characterize Biomphalaria spp. Specimens

Six microsatellite markers were used to characterize *Bi. Pfeifferi*: Bgμl6, Bpf8, rg6, μBG1, U7, and RG9, according to the methodology of Jones et al. (1999) [13,14,15] (Table 1). These loci were purposefully chosen based on their wide-ranging amplicon sizes. The microsatellite markers were pooled into two groups for each DNA specimen in the analysis. Moreover, different dye labels were attached to the forward primers. Then, the samples were sent to Macrogen Europe (https://www.macrogen-europe.com, accessed on 2 December 2022) for fragment analysis. Moreover, two PCR products from each microsatellite marker were produced and, after being purified, were Sanger sequenced, as above, to confirm the amplification of the intended loci. 

### 2.5. Real-Time PCR to Detect Pre-Patent Schistosoma spp. Infections within Snails

Real-time PCR was carried out to detect any pre-patent *Schistosoma* spp. DNA within the *Bi. pfeifferi* extractions. This was conducted by detecting and amplifying a *Schistosoma* spp. genus-specific 77 bp fragment of the internal transcribed spacer-2 (ITS-2) sub-unit, as described previously [8]. The PCR cycling conditions were as follows: 3 min initialization at 95 °C, followed by 50 cycles of 15 s denaturation at 95 °C, 20 s annealing at 60 °C, and 45 s extension at 72 °C. The real-time PCR was conducted on a Chromo4 version 3.1 (Bio Rad, 2015) thermal cycler.

### 2.6. Data Handling and Statistical Analysis

*Cox1* chromatograms were obtained via Sanger sequencing, and the microsatellite data were analyzed using Geneious^®^ (11.1.5). Haplotype network analysis was carried out using PopART software (version 1.7).

## 3. Results

**Figure 2:**
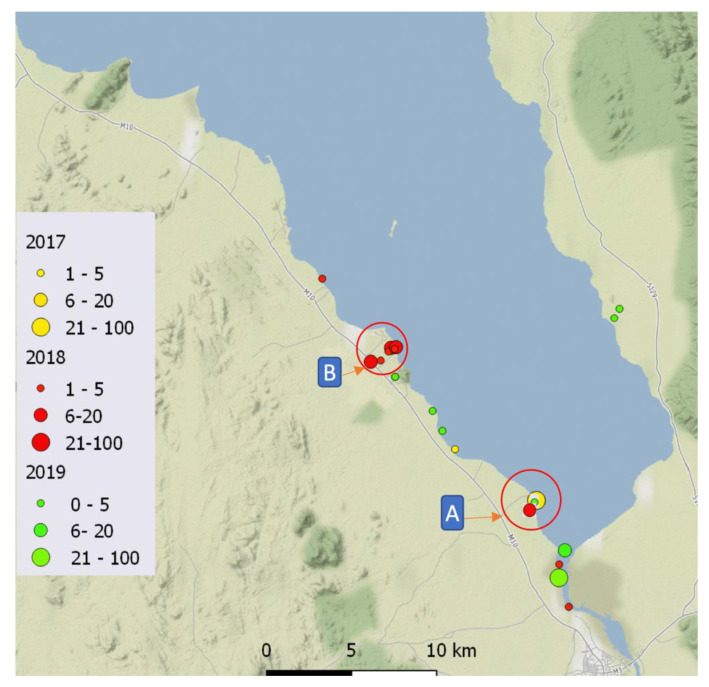
Map (www.qgis.org, accessed on 22 November 2022) shows a summary of colonization sites of *Bi. pfeifferi* in the lake across 2017, 2018, and 2019. (A) Palm Beach, where all *cox*1 haplotypes were identified as present. (B) A small canal irrigation channel leading to the lake from an inland artificial lake fish hatchery.

In total, 201 *Bi. pfeifferi* specimens were collected from 20 locations across the malacological surveys conducted in 2017, 2018, and 2019 (Figure 2). Only one snail was found to be actively shedding *S. mansoni* cercariae in 2018, and just under half of all the collected snails were subjected to all the outlined molecular analyses.

### 3.1. Molecular Characterisation

The Sanger sequence analysis revealed five different haplotypes characterized by five polymorphic sites (Table 2). Of the 76 *Bi. pfeifferi* specimens successfully sequenced, 69 snails were haplotype 1, 5 were haplotype 2, 1 was haplotype 3, and 1 was haplotype 4. Of note, the distribution of haplotype H1 was detected in all the locations surveyed across the three years, whereas haplotypes H2, H4, and H5 were confined to one collection site in 2017 in Palm Beach (Figure 2).

**Figure 3:**
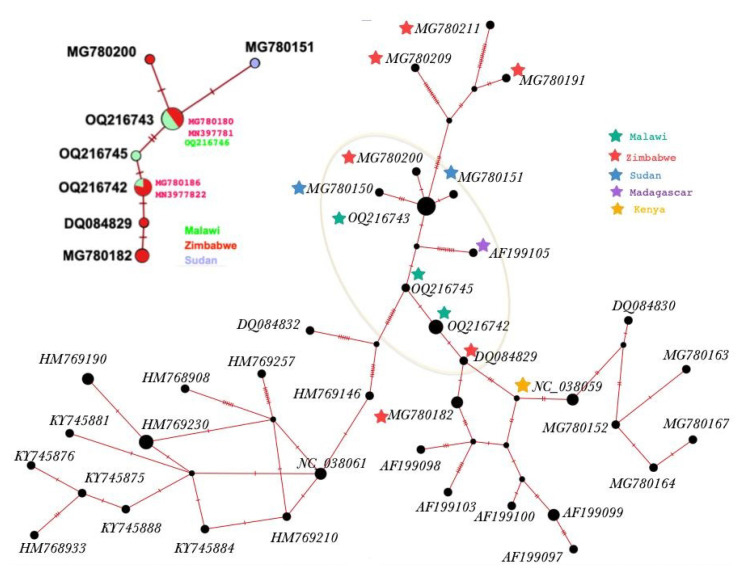
Network of *COX1* gene haplotypes of fifty *Bi. pfeifferi* from GenBank, including the five *cox1* haplotypes from Malawi that were used in this study. In addition, *COX1* haplotype network of samples selected based on genetic pairwise distance to samples from Malawi (similarity > 99%). Hatch mark indicates the different nucleotides in the pairwise alignment.

All of the *cox*1 haplotypes identified in this study (Table 2, Figure 3) are closely genetically related to two haplotypes of *Bi. pfeifferi *identified from Zimbabwe*. *Moreover, the alignments between the *Bi. pfeifferi* samples show the pairwise variation of the haplotypes (Table 2); four SNPs were identified between H1 and H5. 

### 3.2. Microsatellite Data Analysis

**Figure A1:**
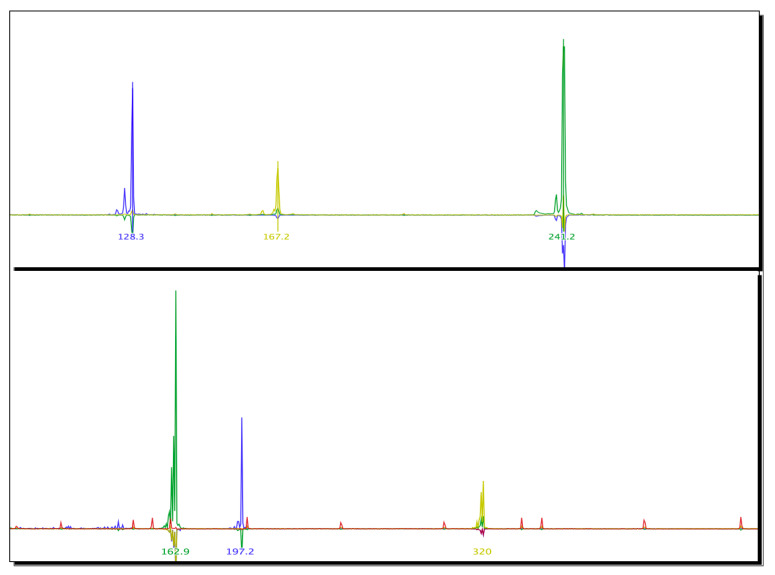
Diagram showing the results of microsatellite data analysis of six loci, indicating the peak of each locus. In order: Bgμl6 (128 bp); Bpf8 (167 bp); rg6 (241 bp); μBG1(162 bp); U7 (197 bp); and RG9 (320 bp).

The microsatellite data analysis showed no variation at these loci. Interestingly, the length of the allele amplicons of Bfp8 and U7 was slightly shorter than expected. Moreover, the examination of the six markers shows that significant heterozygote deficiencies are observed within the sample, as shown by one single peak for each (Figure A1 in Appendix A). The peaks of all the samples showed that all the alleles were homozygous (Table 3). The results of the microsatellite identity analyses were confirmed by sequence analysis via BLAST. 

### 3.3. Detection of *Schistosoma* spp. DNA within Collected *Bi. pfeifferi* Specimens

**Figure 4:**
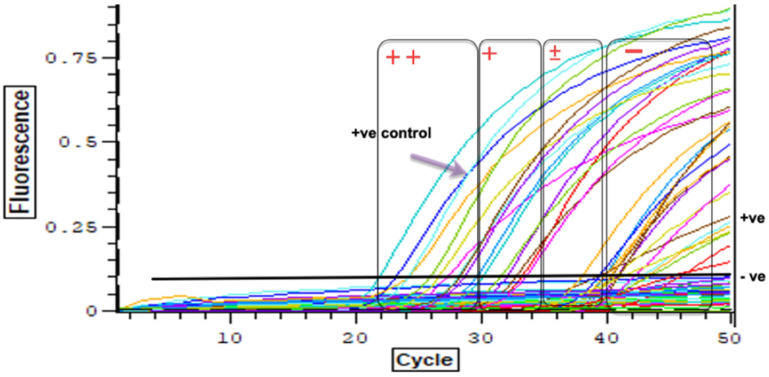
Real-time PCR results show the gradient, strong positive Ct ≤ 30 (++), positive 30 < Ct ≤ 35 (+), trace positive 35 < Ct ≤ 40 (±), negative Ct > 40 (−).

In total, *Schistosoma* DNA was detected within 31% of the examined *Bi. pfeifferi* specimens. Of the 57 snails collected within the lake, only ten samples were infected (13%), whereas 74% of the 19 snails collected from waterbodies nearby the lake were positive by real-time PCR analysis (Table 4) (Figure 4). 

## 4. Discussion

The recent surveys conducted in Malawi since 2017 have revealed significant changes in the epidemiology of schistosomiasis in Malawi, particularly in Lake Malawi, including the first report of *Bi. pfeifferi* [9,10]. The *Biomphalaria* species have exhibited strong local and global spreading capabilities that may advance because of global warming and expansion in the development of the water and agricultural projects [16]. Following a recent invasion and colonization by this species in this area, we report here the invasion patterns and steady expansion of *Bi. pfeifferi* snails from the southern to the eastern region of the lake between 2017 and 2019. In addition, we report, for the first time, a population genetic analysis and dynamic of the transmission of *Bi. pfeifferi* along the southern shoreline of Lake Malawi, Mangochi District. It is worth mentioning that the colonization of *Biomphalaria* was followed by the finding of the *S. mansoni* infection among local people in 2018 [10], before the situation advanced into an outbreak of intestinal schistosomiasis, as reported in 2020 [11]. It is noteworthy that the geographic distribution of *S. mansoni* is reliant on the distribution of the *Biomphalaria* species that play key roles in the parasite transmission [16]. 

The Sanger sequencing and fragment analyses of the *cox*1 gene inferred a genetic lineage of *Bi. pfeifferi* likely originating from Zimbabwe. Furthermore, through microsatellite analyses, five *Bi. pfeifferi* haplotypes were identified in this area. Interestingly, of the five haplotypes, only one haplotype has colonized the lake and water bodies in Mangochi District through 2018 and 2019. Likewise, only Palm Beach contained all five haplotypes in 2017, while all the other sites contained haplotype H1, which is suggestive of a single expansion of snails from Palm Beach with multiple introductions into this particular site. This population of *Bi. pfeifferi* from Lake Malawi has never before been assessed using microsatellite markers, and so, no direct comparisons between these data and other studies have been made until now. 

Our data are in agreement with similar previous studies demonstrating low genetic variation across the *Biomphalaria* populations with these loci [17]. For example, Angers et al. (2003) carried out a genetic study of *Bi. pfeifferi* using Bfp8 loci for samples from various areas: Ivory Coast, Senegal, Ethiopia, Niger and Cameroon, Zimbabwe, Oman, and Madagascar. They found low variation within an individual population, including a population from Zimbabwe, which is further suggestive of an original source being found there. A lack of genetic variation supports our hypothesis that *Bi. pfeifferi* has likely colonized this area of Lake Malawi very recently and has likely driven the recent outbreak of intestinal schistosomiasis locally [11]. In addition, the genetic population structure could have contributed to the dramatic increase in *S. mansoni*, as low genetic variation with putative high schistosome compatibility is known to raise the transmission of *S. mansoni* [18,19,20].

This study has also, for the first time, by way of detecting *Schistosoma* spp. DNA within collected *Bi. pfeifferi* specimens, incriminated the recently established *Bi. pfeifferi* populations in this area as being those actively transmitting intestinal schistosomiasis. In addition, the analysis of these data appears to suggest that transmission of *S. mansoni* is occurring at a higher intensity within the temporal waterbodies surrounding the lake shore than within the lake itself. This may be a result of more frequent human water contact with such waterbodies and may also be because these waterbodies provide a more suitable habitat for *Bi. pfeifferi* populations when compared to the lake. For example, the environmental factors known to hinder *Biomphalaria* population dynamics and population expansion, such as waves and predatory fish, may be lacking within these peripheral waterbodies. It is worthwhile noting that understanding the local biological factors in active transmission areas is essential for snail control [21]. Furthermore, an understanding of the mode of invasion and distribution of *Bi. pfeifferi* in addition to the experimental clarification of the susceptibility to *S. mansoni* would forecast the potential spread of schistosomiasis [16].

The detection of schistosome infections in snails is a promising monitoring tool for successful control and elimination because it helps in the understanding of the local transmission dynamics [22]. However, extra developments are still needed and essential for long-term success [23]. The challenges include the identification of the ideal marker to detect the exact species of schistosome within snails and more field testing alongside a more rigorous standardization of protocols [23]. With the development of molecular methods, several molecular techniques have been used for *Schistosoma* transmission mapping by detecting parasite DNA in snails that were not actively shedding cercariae [22,24,25]. For example, in our study cercarial shedding analyses would have missed new epidemiological developments regarding snail–*Schistosoma* dynamics in the lake without detecting *Schistosoma* infections. In 2022, malacological surveys conducted by a HUGS team in Mangochi District upon examination for shedding cercariae found patently infected Bi. pfeifferi from sites, and upon microscopy these shedding cercariae were confirmed to be of human infecting species, with later ongoing DNA barcoding studies of *S. mansoni* to confirm. Although detecting snails shedding cercariae is the only way to fully incriminate an active intermediate host definitively, a quantitative step has been attempted to facilitate analysis beyond a simple positive or negative result. Nonetheless, there is a lack of standardization in the interpretation of Ct values, which makes the results of real-time PCR assays comparable, although real-time PCR assays can measure relative infection intensities [23]. Although it is a limitation in this study, mapping pre-patent infections in snails provides a promising tool for transmission assessment(s) prior to revision of the current control and future prevention. We recommend the expansion of future malacological surveillance with expanded molecular xenomonitoring to better monitor the environmental epidemiology. 

In conclusion, this study aimed to understand the dynamics, molecular identification, and genetic structure of *Biomphalaria*, alongside detection of the schistosome parasites within the snails. We observed a low genetic diversity of *Bi. pfeifferi*, which might enhance the transmission of intestinal schistosomiasis. Looking to the future, we expect larger zones of the lake to be at risk of intestinal schistosomiasis, which calls for closer epidemiological and malacological surveillance, with future molecular xenomonitoring inspections.

## Figures and Tables

**Table 1:** Primers used for six chosen microsatellite marker loci (Jones et al. 1999).

Locus	Primer Sequence (5′-3′)	Source
Bpf8	F:GGTTCCCATAGCATACAGTGC R:GGCTTACAAAGAAACAGGCATAC	[13]
U-7	F: TCATGACATCTAATGGGAGAAG R:GGGCTCAGAGAAATGAATGG	Unpublished data
rg6	F: GATTTTGTCTCACGGAAACG R:GCGTGCTTATGTAGCAAAGG	[14]
rg9	F:GGAGCTGTCGTAATTATAGTTCA R:TTGAGGTGTCATGGTTCTAGG	[14]
Bgµ16	F:CTGTTATTCATTATTTCATAGAGC R: GGGGATCTAACACATCAG	[15]
µBg1	F:TTAATTCTACTGGACTCACATGG R:CTGCCAATGTTTACATGCTG	[15]

**Table 2:** Nucleotide variation within each of the *cox*1 haplotypes against NC_038059 from GenBank, which was used as a reference DNA sequence for comparisons. The alignments between *Bi. pfeifferi* show five single nucleotide polymorphism (SNP) point mutations on the mitochondrial *cox*1 gene of *Bi. pfeifferi* at positions 169, 187, 250, 537, and 569.

Haplotype & Acc.No	169	187	250	537	569
**NC_038059**	T	G	A	G	G
**H1 (OQ216742)**	T	G	A	G	G
**H2 (OQ216743)**	C	A	G	G	G
**H3 (OQ216744)**	C	A	G	T	G
**H4 (OQ216745)**	T	A	A	G	G
**H5 (OQ216746)**	C	A	G	G	T

**Table 3:** Details six microsatellite markers, size of amplicons, and allele frequency.

N	Marker	Expected Amplicon Size Range (bp)	Size of Amplicon	Allele Frequency
1	Bgμl6	113–135	128 bp	1.00
2	Bpf8	193–225	167 bp	1.00
3	rg6	233–244	241 bp	1.00
4	μBG1	165–167	162 bp	1.00
5	U7	222–246	197 bp	1.00
6	RG9	313–321	320 bp	1.00

**Table 4:** Summary of collection data and molecular investigation of* Bi. pfeifferi*.

Location	Type	Year	Latitude	Longitude	Number of Snails Examined	Infected	Haplotype and Acc.No
1	Lake	2017	−14.36919	35.17629	2	0	H1 OQ216742
2	Lake		−14.39363	35.22104	17	2	H1 OQ216742 H2 OQ216743 H3 OQ216744 H4 OQ216745 H5 OQ216746
3	Lake	2018	−14.27752	35.10419	1	0	H1 OQ216742
4	Stream		−14.32120	35.13072	5	4	
5	Lake		−14.39363	35.22104	22	5	
6	Lake		−14.44975	35.24018	3	3	
7	Lake		−14.42708	35.23349	1	0	
8	Stream		−14.31371	35.14174	4	4	
9	Stream		−14.31568	35.14030	2	2	
10	Stream		−14.32033	35.13613	2	1	
11	Stream		−14.31338	35.14449	1	0	
12	Lake		−14.31437	35.14376	2	0	
13	Lake	2019	−14.44930	35.23871	1	0	
14	River		−14.34131	35.28897	2	1	
15	River		−14.41937	35.23394	2	1	
16	River		−14.32895	35.14481	1	1	
17	Lake		−14.39545	35.22569	3	0	
18	Lake		−14.29649	35.25654	2	0	
19	Lake		−14.31494	35.26697	2	0	
20	Lake		−14.36913	35.17623	1	0	

## Data Availability

Not applicable.
